# Supplementary material for: AnnoMiner is a new web-tool to integrate epigenetics, transcription factor occupancy and transcriptomics data to predict transcriptional regulators
Source: Sci Rep. 2021 Jul 29;11:15463. doi: 10.1038/s41598-021-94805-1 (PMC8322331; doi:10.1038/s41598-021-94805-1)
Supplement: Supplementary file 1 — Supplementary Information 1. [file 41598_2021_94805_MOESM1_ESM.pdf]

AnnoMiner is a new web-tool to integrate epigenetics, transcription factor occupancy and transcriptomics data to predict transcriptional regulators

Arno Meiler[1*], Fabio Marchiano[2*], Michaela Weikunat[1], Frank Schnorrer[1,2] and Bianca H. Habermann[1,2]

1 Max Planck Institute of Biochemistry, Am Klopferspitz 18, 82152, Martinsried, Germany
2 Aix-Marseille University, CNRS, IBDM UMR 7288, The Turing Center for Living Systems, 13009 Marseille, France
* these authors contributed equally

Supplementary Data

Supplementary Tables

Supplementary Table S6: Fly strains used and their observed phenotypes

Genotype	lethality	flight
Mef2-GAL4; UAS-GFP-Gma / +	viable	wild type
Mef2-GAL4; UAS-GFP-Gma / Trl-IR-1 (VDRC KK106433)	viable	flightless
Mef2-GAL4; UAS-GFP-Gma / Trl-IR-2 (VDRC GD41095)	pupal lethal	-
Mef2-GAL4; UAS-GFP-Gma / Trl-IR-3 (BDSC HMS02188)	viable	flightless
Mef2-GAL4; UAS-GFP-Gma / Trl-IR-4 (VDRC GD17189)	pupal lethal	-
Mef2-GAL4; UAS-GFP-Gma / CG14655-IR-1 (VDRC 13136)	viable	flightless
Mef2-GAL4; UAS-GFP-Gma / CG14655-IR-2 (VDRC 104293)	pupal lethal	-
Mef2-GAL4; UAS-GFP-Gma / CG14655-IR-3 (BDSC JF02334)	pupal lethal	-

Supplementary Table S8: Genome assemblies available in the AnnoMiner web-server

Assembly	Ensembl	Refseq	Ucsc	Genecode	Wormbase	Flybase
**hg38**		**√**	**√**	**√ (34)**		
**hg19**	**√**	**√**	**√**	**√ (34lift37)**		
**mm10**		**√**	**√**	**√ (M25)**		
**mm9**	**√**	**√ (no mT)**	**√**			
**dm6**	**√**	**√**				
**dm3**	**√**	**√ (no mT)**				**√**
**ce11**	**√**	**√**			**√ (WS245)**	
**sacCer3**	**√**	**√**				

Supplementary Table S9: Available TF ChIP-seq data for all model organisms and genome assemblies

Assembly	TF-ChIP-seq data available	unique TFs
**hg19**	1687	666
**hg38**	1586	660
**mm10**	154	47
**dm6**	514	474
**dm3**	360	325
**ce11**	468	286

Supplementary Figures
Supplementary Figure S1

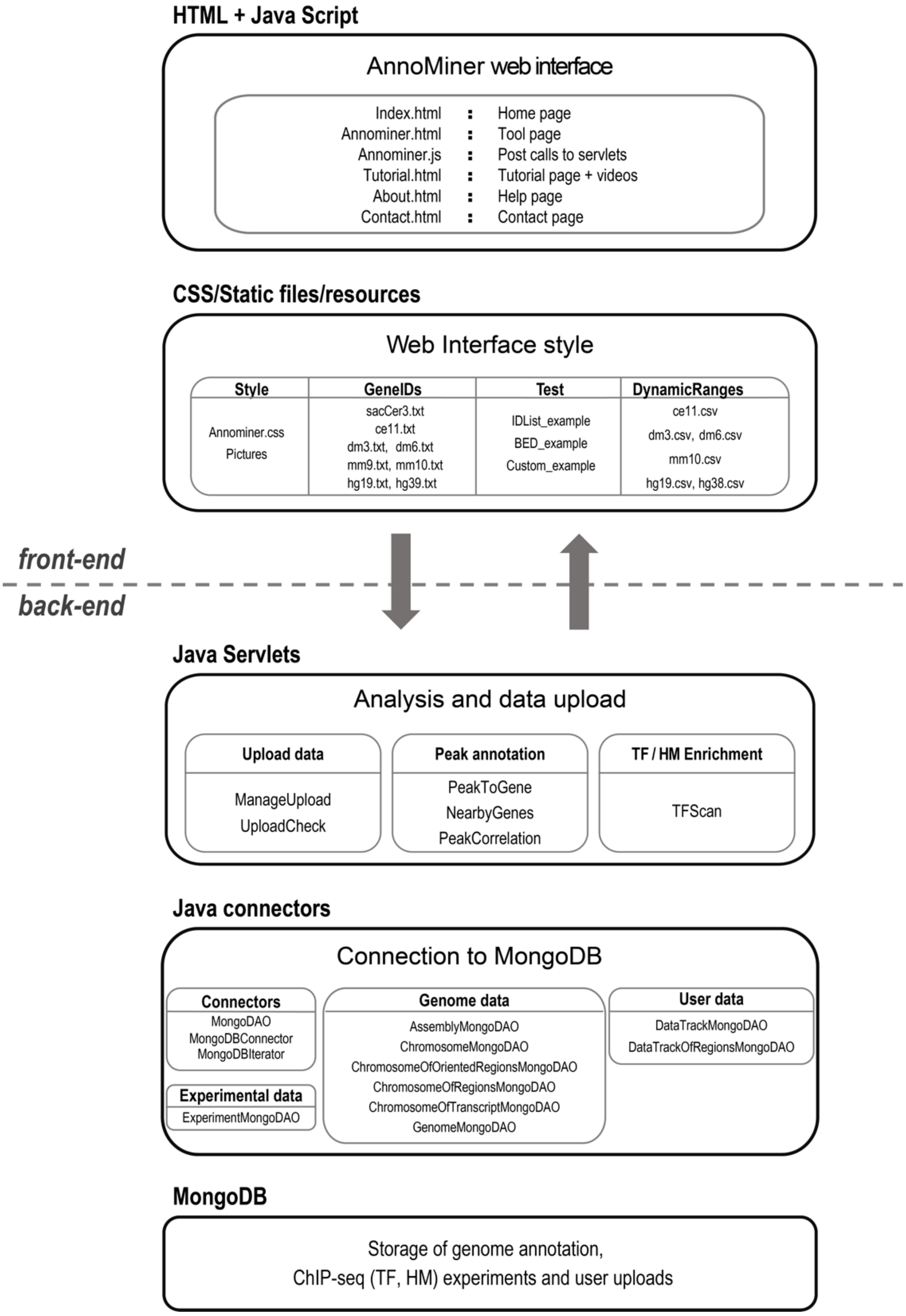

Figure legend for Supplementary Figure S1: Back- and front-end structure of the AnnoMiner web-platform. In the back-end, the non-relational MongoDB database stores the annotated genomes, as well as the experimental epigenetic data (ChIP-seq data of transcription factors and histone modifications) that are publicly available via ENCODE, modENCODE and modERN. User-uploaded data are stored temporarily and are only available to the user. A set of java scripts connect to the MongoDB database for genome annotation and data retrieval. Different java servlets are created to carry out multiple functions in AnnoMiner: ManageUpload/UploadCheck to manage and store user data

in the MongoDB; PeakToGene/NearbyGenes/PeakCorrelation to perform peak annotation and integration analysis; and TFScan to perform transcription factor (TF)/histone mark (HM) enrichment analysis functions. The front-end is made of static html pages where a main javascript file handles the DOM events, parameter choices and data visualizations. Among the static files are the GeneIDs files used for the gene id converter and the ones for the DynamicRanges which is needed by the TFScan function to dynamically assess the upstream promoter boundaries for each TF. Both these files are easily accessible and in plain text format in order to allow easy update and/or modification by the user. The interface appearance is set by the CSS layer (mainly Bootstrap 4). Finally, test files are provided, which together with the tutorial page and video tutorials aim to quickly get the user started in using AnnoMiner. To make AnnoMiner even more user-friendly, we have created tutorial videos, explaining in detail its usage, available at: http://chimborazo.ibdm.univ-mrs.fr/AnnoMiner/tutorial.html

Supplementary Figure S2

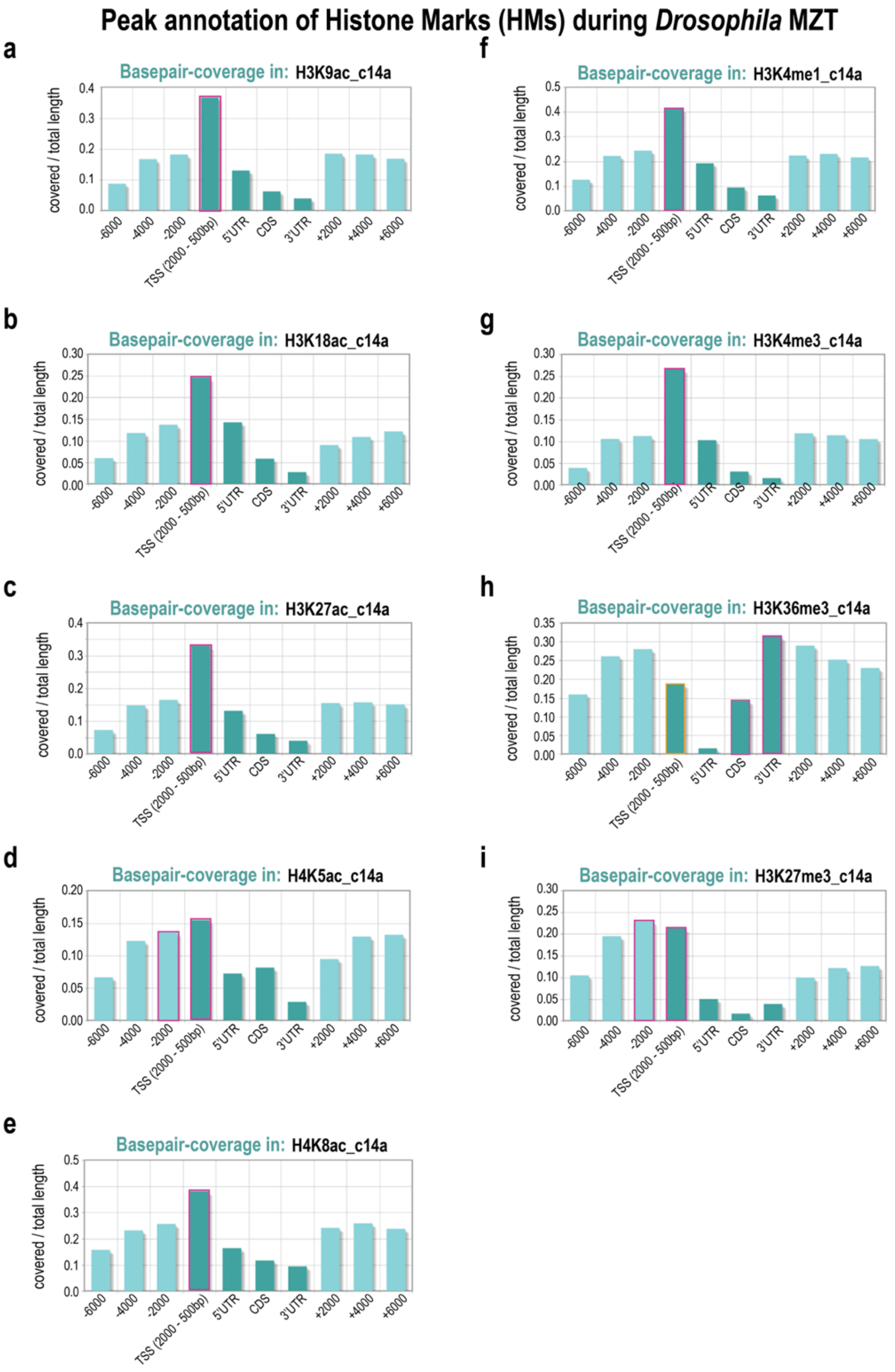

Supplementary Figure S2: AnnoMiner *peak annotation* with different histone modifications (HMs) during *Drosophila* maternal-to-zygotic transition (MZT). Li et al. systematically analysed different types histone modifications during MZT, aiming to identify regions of genomic activity during this phase of early development in *Drosophila* [1]. Shown are activating **(a-g)**, as well as gene body **(h)**, and repressing **(i)** histone modifications. Red rectangles around bars indicate chosen elements for gene annotation. For the gene body histone modification H3K36me3, two regions were chosen: gene body (red rectangle), as well as TSS alone (orange rectangle), resulting in two different gene lists. We chose

nuclear cycle (nc) 14a, which is the time-point representing a late transition stage between maternal RNAs and zygotic RNAs. Associated gene lists are given in Supplementary Table S1 a-j. Original peak data from [1], deposited in GEO dataset GSE58935, were taken for upload to AnnoMiner. Genome version dm3 was used in AnnoMiner for peak annotation, together with the refseq database.

Supplementary Figure S3

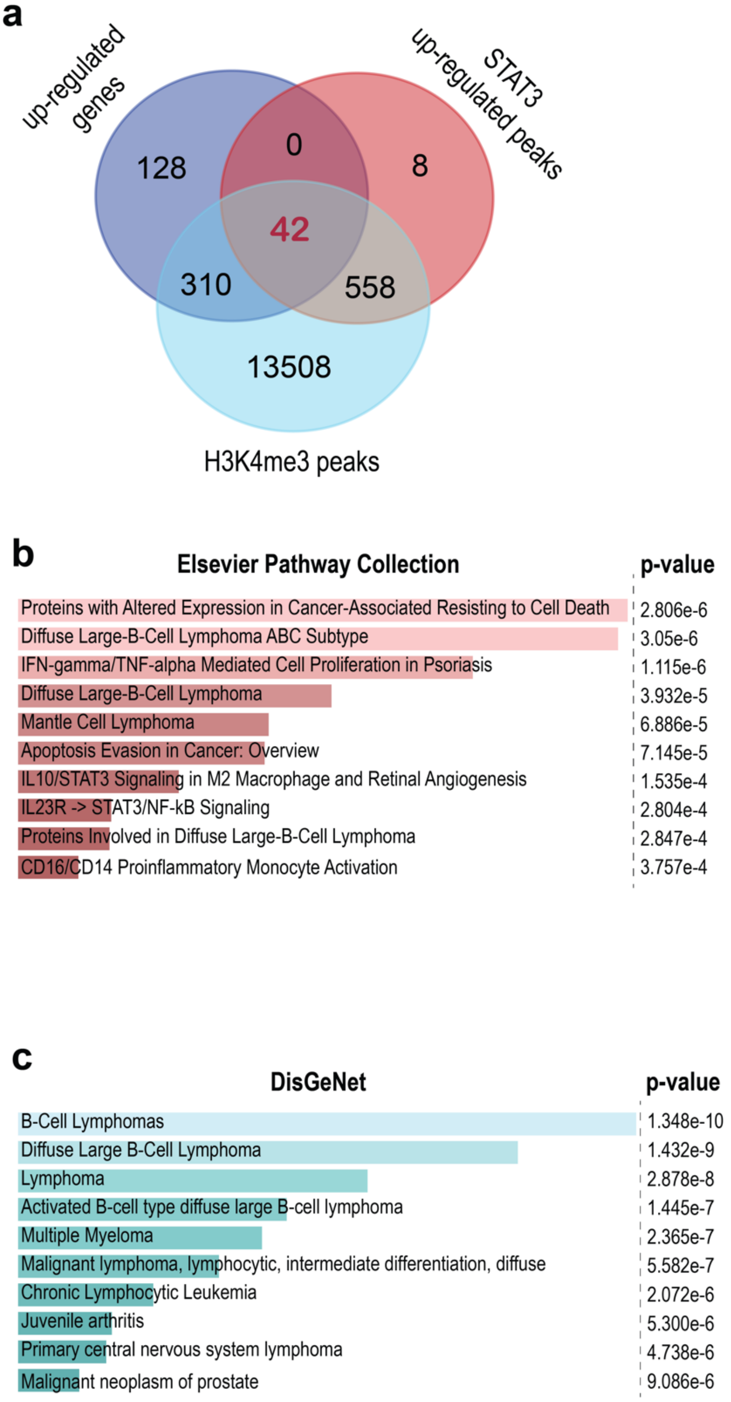

Figure legend for Supplementary Figure S3: Data integration of STAT-3 upregulated peaks in different types diffuse large B-cell lymphomas (DLBCL) with H3K4me3 methylation status as well as differential expression data. TF data of STAT3 from two forms of DLBCL, the more aggressive activated B-cell like (ABC) with higher levels of STAT3 expression and the germinal center B-cell like (GBC) (GEO dataset GSE50723 from super-series GSE50724, 10.1534/g3.113.007674) were compared. Only peaks that were significant and upregulated in ABC with an FDR of <0.05 and a log2FC of >0.31 (corresponding to a fold change of >1.25) were chosen for upload. The major peak at the TSS shown in Figure 4 from the main text was chosen for further analysis. H3K4me3 methylation data from

DLBCL cell line Ly3 was chosen to integrate STAT3 data with open chromatin status (GEO dataset GSE86718). Again, the significant peak at the TSS was chosen for further analysis (main Figure 4). **(a)** STAT3 upregulated peaks were integrated with H3K4me3 peaks, as well as upregulated genes in ABC subtype (taken from GEO dataset GSE50721 from super-series GSE50724). 42 genes were upregulated and had both, an upregulated STAT3 peak in ABC subtype and open chromatin in the cell line Ly3. These can be considered direct target genes of STAT3. **(b)** EnrichR enrichment analysis of these 42 intersecting genes revealed strong enrichment of pathway terms related to 'Proteins with Altered Expression in Cancer-Associated Resisting to Cell Death', 'Diffuse Large-B-Cell Lymphoma ABC Subtype', 'Apoptosis Evasion in Cancer; Overview' or 'IL10/STAT3 Signaling in M2 Macrophage and Retinal Angiogenesis', just to name a few. **(c)** DisGeNet enrichment using EnrichR revealed equally relevant terms, including 'B-Cell Lymphomas', 'Diffuse Large-B-Cell Lymphoma', Activated B-cell type diffuse large B-cell lymphoma', among others. All data on upregulated genes associated with STAT3, H3K4me3 or both, as well as enrichment results are available in Supplementary Table S2.

Supplementary Figure S4

Peak integration of H3K4me3 during 4 stages of *Drosophila* MZT

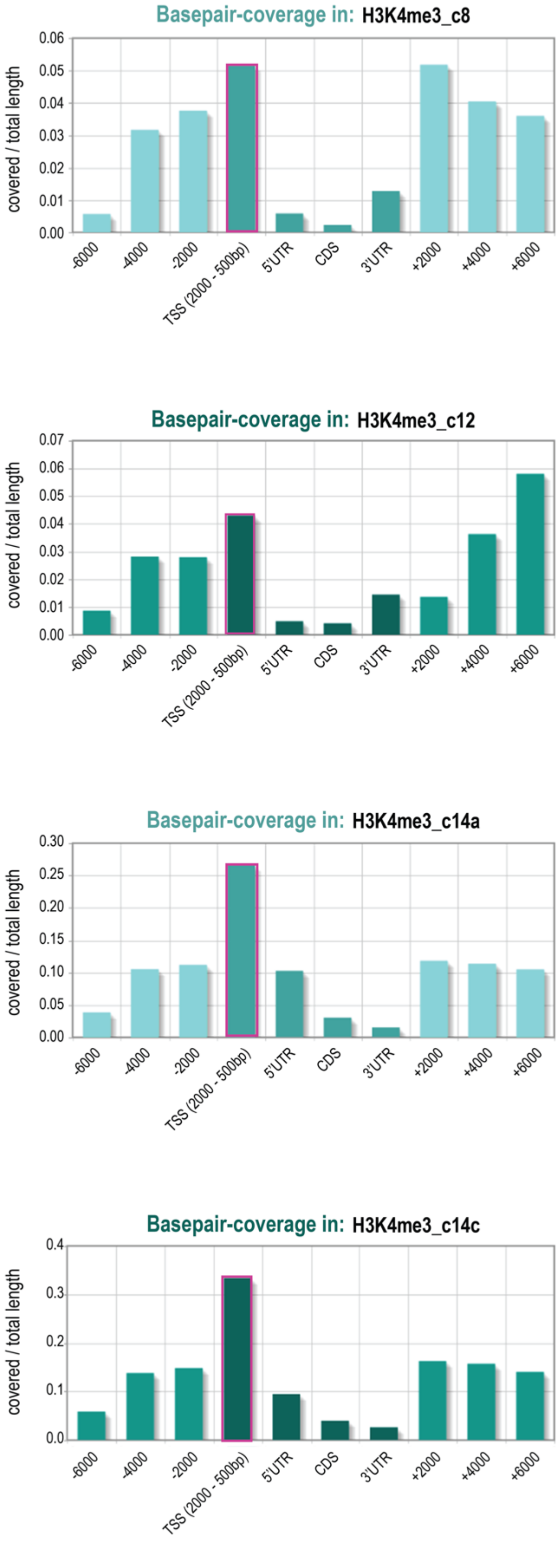

Supplementary Figure S4: Peak integration of H3K4me3 during 4 stages of Drosophila maternal-to-zygotic transition (MZT). To demonstrate AnnoMiner's peak integration function with several datasets, we chose a time-series of histone modifications from the MZT phase of early Drosophila

development [1]. H3K4me3 methylation becomes highly enriched in late stages of MZT (c14a), however it is also found in some promoters during stages c8 and c12. We uploaded peak data of H3K4me3 methylation from all 4 stages (c8-c14c) from GEO dataset GSE58935 to AnnoMiner and performed peak integration on all 4 bed files, using Drosophila genome version dm3. Extracted genes can be found in Supplementary Table S1 k. We found 19 genes with constitutive presence of H3K4me3 in their promoter, including the transcriptional regulator modifier of mdg4 (mod(mdg4)), RNA binding and splicing factors such as Pcf11, CG7879 and scaf6, and most importantly smaug, which is a sequence-specific RNA-binding protein involved in inhibition of translation and induction of degradation of hundreds of maternal mRNAs during MZT in early embryos.

Supplementary Figure S5

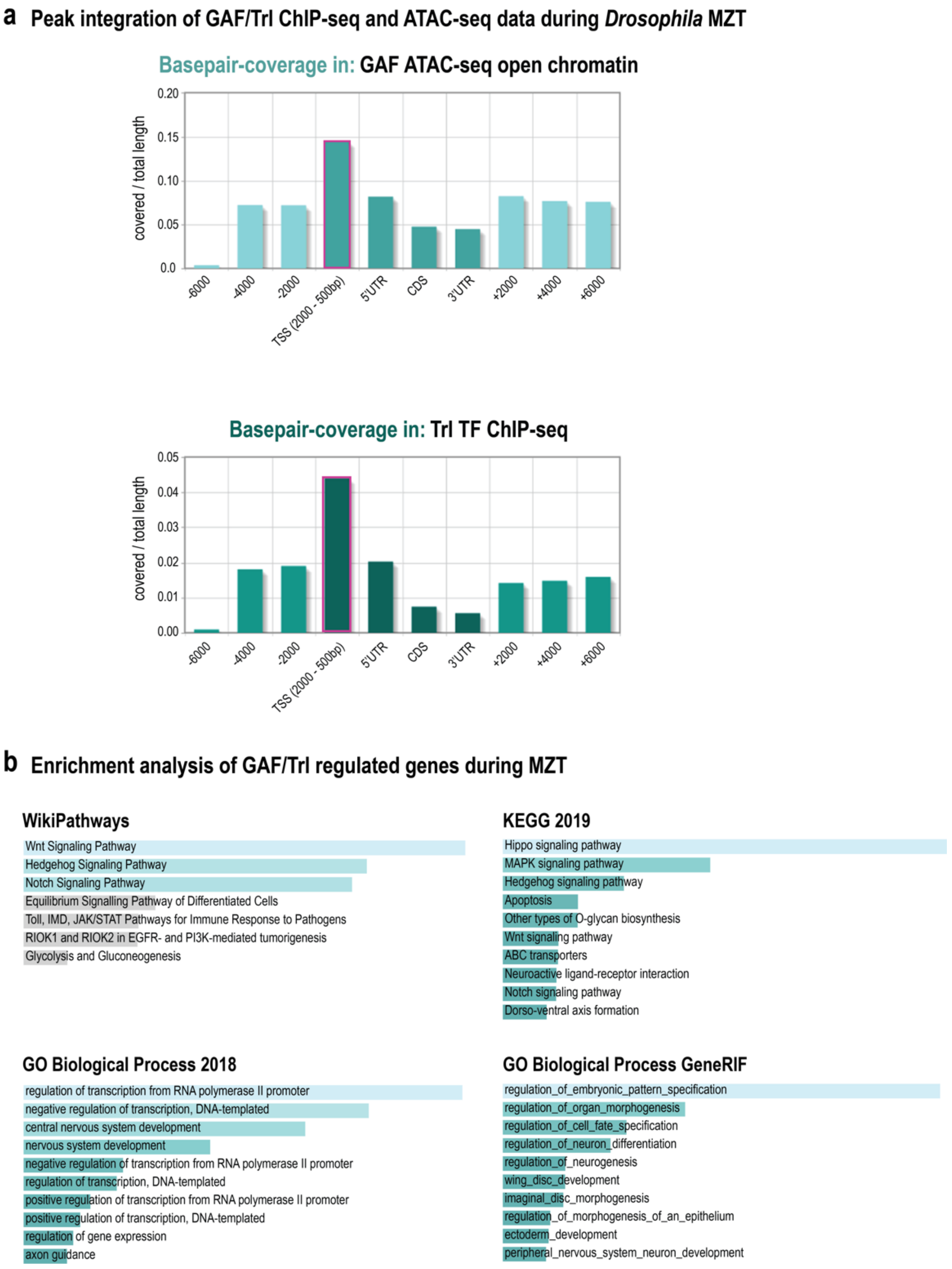

Supplementary Figure S5: Peak integration of ATAC-seq and GAF/Trl transcription factor ChIP-seq data to identify genes regulated by GAF during later stages of MZT in *Drosophila*. To further demonstrate AnnoMiner's peak integration function, we chose data looking at GAF/Trl-dependent transcriptional activation necessary for zygotic genome activation during MZT in *Drosophila* [2]. **(a)** We took ChIP-seq TF data from GAF/Trl, ATAC-seq, as well as RNA-seq data from GEO series GSE152773. Bed-files from ChIP-seq and ATAC-seq were taken as is, RNA-seq count data from GAF degraded versus wild-type were analysed for differential expression using DESeq2 [3]. Bed files were uploaded to AnnoMiner, together with the GAF annotation file containing data from differential

expression analysis. The prominent peaks of GAF ATAC-seq, as well as GAF/Trl ChIP-seq data were selected and genes harbouring peaks of both factors in their TSS region were extracted (Supplementary Table S3). 642 transcripts from 250 unique genes were found to be potentially regulated by GAF/Trl and containing an open chromatin according to ATAC-seq data. **(b)** We performed enrichment analysis of genes annotated with AnnoMiner and found predominantly signalling pathways involved development enriched, as well as biological processes related to embryonic development, organ morphogenesis, transcriptional regulation as well as many other terms related to developmental and cell differentiation processes (see also Supplementary Table S3).

Supplementary Figure S6

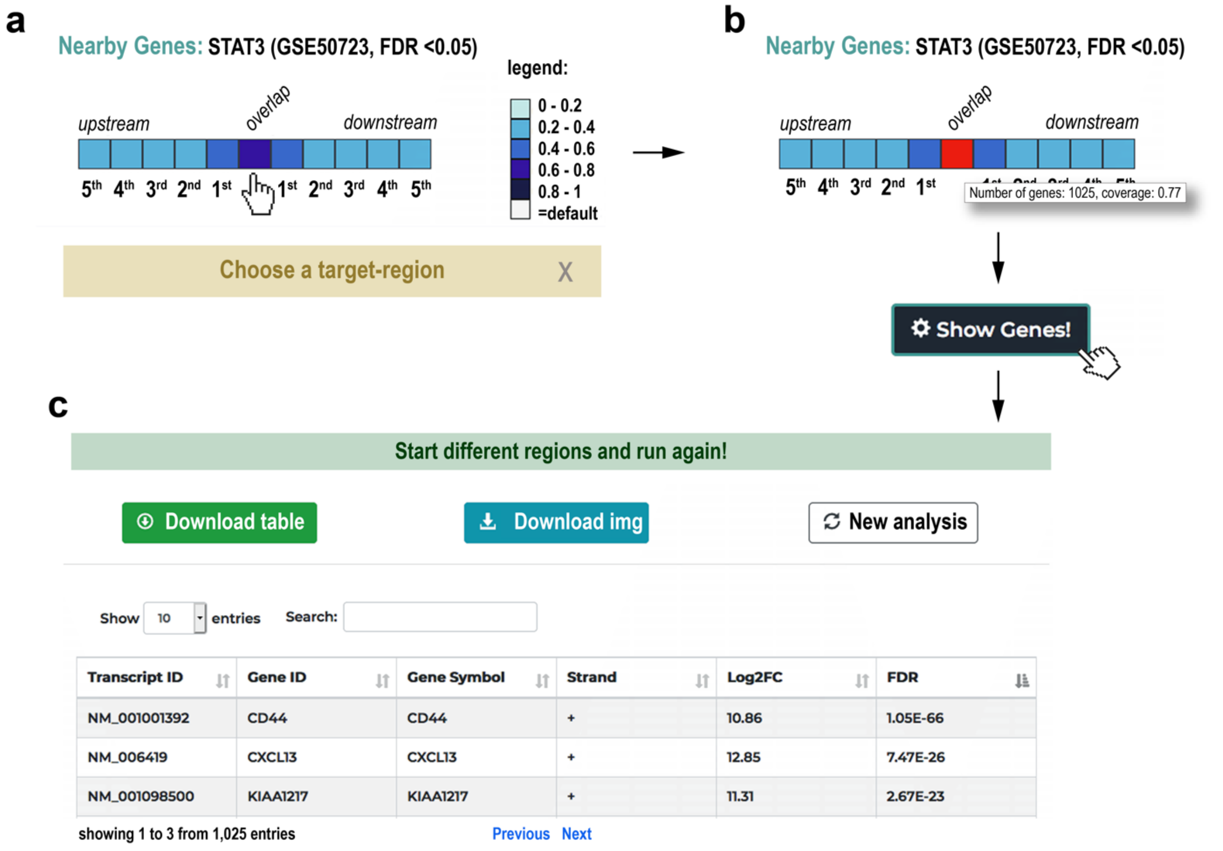

Figure legend for Supplementary Figure S6: The nearby gene annotation function applied for multiple peaks. The nearby gene function can be also used to explore the correlation of multiple peaks of a TF with their 10 neighbouring genes. **(a)** The single-lane box plot shows the overlapping genes as well as the 5 upstream and 5 downstream neighbouring genes of all peaks from the submitted peak file. The colouring corresponds to the % deregulated genes at a certain position relative to the peaks. **(b)** The user can select a target region (here the overlapping genes), which pops up the information of associated genes and which percentage of those are deregulated. **(c)** When clicking on the 'Show Genes!' button, a table is shown with all deregulated genes of the selected region. Significant differential peaks from STAT3 (GEO dataset GSE50723) as well as associated differential expression data (GEO dataset GSE50721, both from super-series GSE50724) were uploaded for producing this Supplementary Figure.

Supplementary Figure S7

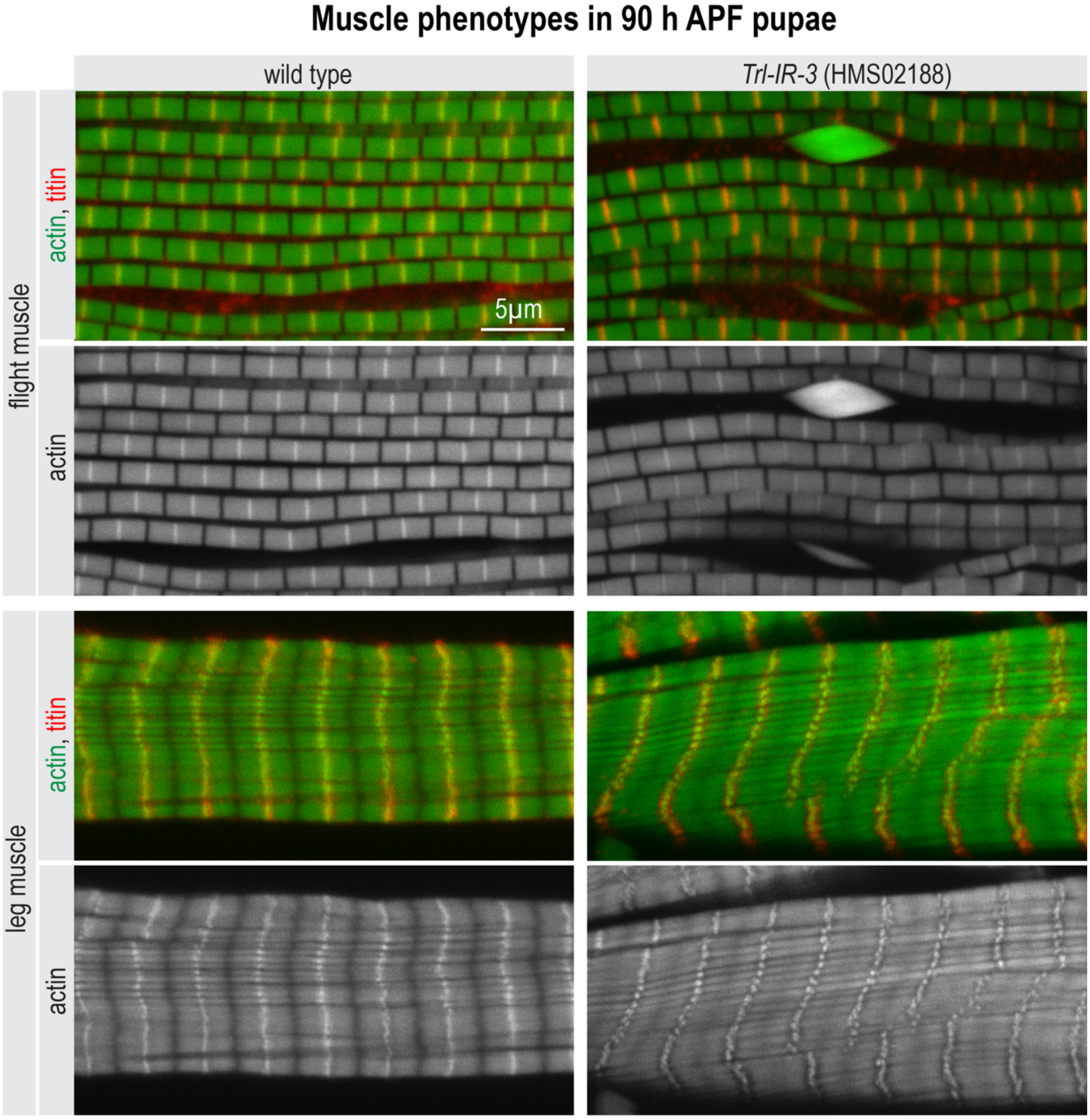

Figure legend for Supplementary Figure S7: *Trl* has a function during flight muscle myofibrillogenesis. Flight and leg muscles of 90 h APF wild type or *Trl-IR-3* pupae were fixed and stained for actin (phalloidin in green) and titin homolog Sls (anti-Kettin in red). Note the abnormal actin accumulations in *Trl-IR-3* flight muscles compared to wild type.

Supplementary Figure S8

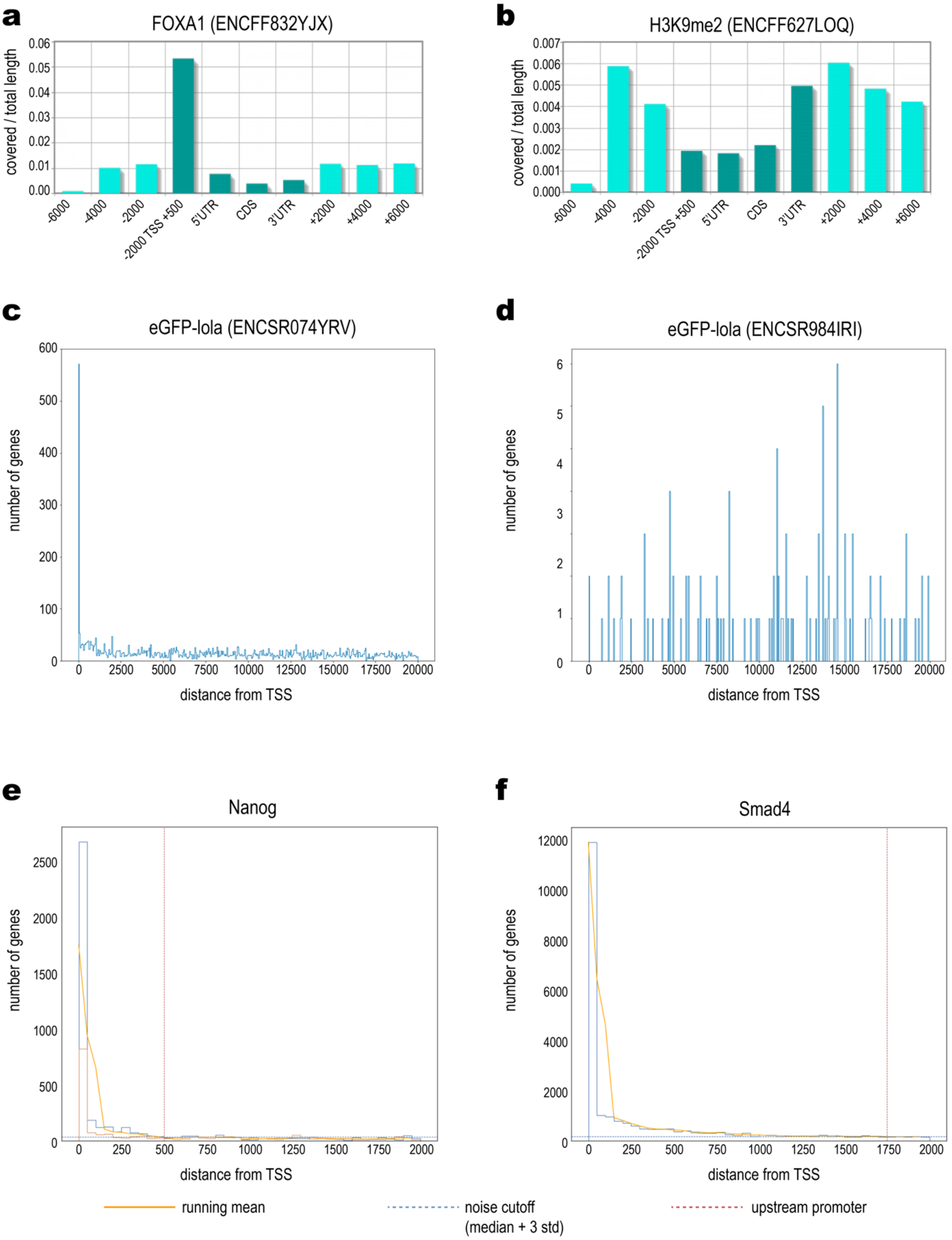

Figure legend for Supplementary Figure S8: Coverage profiles of transcriptional regulators. (a) Coverage profile of the transcription factor FOXA1 (conservative IDR thresholded peaks downloaded from data set ENCSR735KEY, *H. sapiens* liver from a male adult, taken from the ENCODE database). A clear peak in the promoter region, defined as Transcription Start Site (TSS), flanked by -2000bp and +500bp respectively, can be seen. **(b)** Coverage profile of the transcriptional regulator TP53

(conservative IDR thresholded peaks taken from ENCODE data set ENCSR980EGJ, *H. sapiens* HepG2 cells). TP53 has a broader distribution, ranging from the TSS to -4000 bp upstream, as well as shows significant overlap with the 3' downstream region of gene features. **(c)** Regular (ENCSR074YRV) and **(d)** outlier (ENCSR984IRI) distribution of the *Drosophila* transcriptional regulator Lola, available for dm6, Refseq. Distances between each peak and genes in a range of 20,000 bp upstream of their TSSs were computed, and then binned with a resolution of 50 bp. While ENCSR074YRV **(c)** clearly shows preferential binding close to genes TSS, ENCSR984IRI **(d)** shows few and non-preferential bindings. Therefore, it was marked and excluded as outlier. **(e)** Cumulative plot of the Nanog transcription factor over all uploaded data sets. Nanog's running mean drops below the noise level at -500 bp upstream the TSSs of associated gene features. **(f)** Cumulative plot of the Smad4 transcription factor over all uploaded data sets. The running mean of Smad4 drops below the noise level only at -1750bp upstream of the TSSs of associated gene features. **(a + b)** Coverage is expressed as base pairs covered / total length.

Supplementary References

1. Li, X.-Y., Harrison, M. M., Villalta, J. E., Kaplan, T. & Eisen, M. B. Establishment of regions of genomic activity during the Drosophila maternal to zygotic transition. *Elife* **3,** (2014).
2. Gaskill, M. M., Gibson, T. J., Larson, E. D. & Harrison, M. M. GAF is essential for zygotic genome activation and chromatin accessibility in the early Drosophila embryo. *Elife* **10,** (2021).
3. Love, M. I., Huber, W. & Anders, S. Moderated estimation of fold change and dispersion for RNA-seq data with DESeq2. **15,** 550–21 (2014).
